# Divergent effects of brief contemplative practices in response to an acute stressor: A randomized controlled trial of brief breath awareness, loving-kindness, gratitude or an attention control practice

**DOI:** 10.1371/journal.pone.0207765

**Published:** 2018-12-12

**Authors:** Matthew J. Hirshberg, Simon B. Goldberg, Stacey M. Schaefer, Lisa Flook, David Findley, Richard J. Davidson

**Affiliations:** 1 Center for Healthy Minds, University of Wisconsin-Madison, Madison, WI, United States of America; 2 Department of Educational Psychology, University of Wisconsin-Madison, Madison, WI, United States of America; 3 Department of Counseling Psychology, University of Wisconsin-Madison, Madison, WI, United States of America; 4 VA Puget Sound Health Care System-Seattle Division, Seattle, Washington, United States of America; 5 Department of Psychology, University of Wisconsin-Madison, Madison, WI, United States of America; Chinese University of Hong Kong, HONG KONG

## Abstract

Mindfulness practices are increasingly being utilized as a method for cultivating well-being. The term mindfulness is often used as an umbrella for a variety of different practices and many mindfulness-based interventions (MBIs) contain multiple styles of practice. Despite the diversity of practices within MBIs, few studies have investigated whether constituent practices produce specific effects. We randomized 156 undergraduates to one of four brief practices: breath awareness, loving-kindness, gratitude, or to an attention control condition. We assessed practice effects on affect following brief training, and effects on affect and behavior after provocation with a stressor (i.e., Cold pressor test). Results indicate that gratitude training significantly improved positive affect compared to breath awareness (*d* = 0.58) and loving-kindness led to significantly greater reductions in implicit negative affect compared to the control condition (*d* = 0.59) immediately after brief practice. In spite of gains in positive affect, the gratitude group demonstrated increased reactivity to the stressor, reporting the CPT as significantly more aversive than the control condition (*d* = 0.46) and showing significantly greater increases in negative affect compared to the breath awareness, loving-kindness, and control groups (*d*s = 0.55, 0.60, 0.65, respectively). Greater gains in implicit positive affect following gratitude training predicted decreased post-stressor likability ratings of novel neutral faces compared to breath awareness, loving-kindness, and control groups (*d*s = - 0.39, -0.40, -0.33, respectively) as well. Moreover, the gratitude group was significantly less likely to donate time than the loving-kindness group in an ecologically valid opportunity to provide unrewarded support. These data suggest that different styles of contemplative practice may produce different effects in the context of brief, introductory practice and these differences may be heightened by stress. Implications for the study of contemplative practices are discussed.

## Introduction

Contemplative practices are increasingly being used as a means for enhancing well-being. In particular, mindfulness has been promoted as a method for enhancing attention, reducing stress, and increasing physical and mental health. Mindfulness is commonly defined as paying attention to present-moment experience, on purpose, with an attitude of acceptance or non-judgment [1]. A growing corpus of empirical research suggests that quality mindfulness-based interventions (MBI) are effective in reducing a variety of mental health disorders, including anxiety and depression, managing physical pain symptoms, supporting substance abuse recovery, and promoting well-being [e.g., 2, 3].

Most MBIs are derivatives of the paradigmatic Mindfulness-Based Stress Reduction program (MBSR) [4]. MBSR practices originate primarily from Buddhist and yogic meditative techniques [4]. MBSR and its derivatives are not comprised of a single practice or even a single style of practice. Rather, MBSR contains at least four main techniques (i.e., awareness of breath, awareness of body sensations, walking meditation, mindful movement). A fifth technique, loving-kindness practice, is taught during the MBSR day of practice that occurs around week six or seven in the 8-week program. Many contemporary MBSR classes as well as related MBIs now integrate loving-kindness as a core component throughout [5]. Research on MBSR and MBIs generally present program effects as mindfulness effects, but in Buddhist meditation taxonomies, some constituent MBI practices are understood to produce different and specific practice outcomes. In particular, outcomes from loving-kindness and breath awareness are expected to be different [6, 7].

Relatively little attention has been paid to the possibility that constituent MBI practices may produce divergent effects. Attending to the specificity of practice type outcome is important for operationalizing mindfulness, for the inferences drawn from MBIs, and for informing maximally efficacious interventions. The few studies that have examined the impact of an isolated practice common in MBIs indicate that attending to the specificity of practice effects is warranted. For example, brief loving-kindness practice produced higher positive affect and more positive explicit and implicit evaluations of others [8]. Compared to sham mindfulness or no training, brief breath awareness training reduced negative affect and heart rate while increasing mood [9]. In a comparative study of three-months of practice in two styles of practice, but not two discrete practice techniques, heart rate, subjective effort, and the degree to which participants liked the practice significantly differed between styles of practice emphasizing calmly abiding attention (to breath or body sensations) and practices designated as socio-affective (e.g., loving-kindness: cultivating feelings of warmth and goodwill to one’s self and others) [10].

As research on mindfulness and other contemplative practices matures, comparisons among MBI practices and between these and other forms of contemplation are needed. Assessing possible outcome divergences in the context of a stressor is particularly salient. As the name Mindfulness-Based Stress Reduction intimates, the paradigmatic MBI was initially developed to reduce subjective experiences of stress as a result of chronic pain [5]. Strategies that promote adaptive stress regulation are important because stress degrades top-down regulatory processes, promotes negative affect, and biases behaviors [11]. For example, stress and negative affect bias first impressions [12] and lead to lower likability ratings of emotionally ambiguous faces [13]. Individual experiences of stress, as opposed to stress experienced collectively as part of a group, attenuate generous behaviors [14]. Thus, there are individual (e.g., increased negative affect) and social (e.g., reduced generosity) consequences of stress. One way mindfulness practice is initially thought to reduce the deleterious consequences of stress is by promoting top-down regulatory processing with salutary effects on affect and behavior [15, 16]. But because MBIs are comprised of multiple practices, it is not clear whether certain techniques contribute more to adaptive stress regulation or whether consistent mechanisms are active across practices or levels of contemplative experience.

Despite the potential importance of understanding the impact of constituent mindfulness practices in the context of an acute stressful event, we are not aware of a study that has examined this possibility. In the study cited above comparing effects of practice style over three months of training, practicing mindfulness reduced the subjective negativity of a laboratory stressor but did not impact stress physiology (e.g., cortisol secretion). However, physiological responses to stress were reduced by loving-kindness and compassion practices and socio-cognitive techniques (e.g., involving perspective-taking [17]). These compelling results do not isolate the effects of a specific practice technique, however, and occurred after several months of practice. Popular mindfulness apps (e.g., Headspace, 10% Happier) involve short daily doses (i.e., < 15 minutes) often over short durations. Virtually nothing is known about whether a brief introduction to one contemplative practice or another might differentially affect reactivity to an acute stressor. The present study was designed to address this gap in the literature.

We compared the effects of three brief contemplative practices and one control mental exercise in response to an acute laboratory stressor. Two of the contemplative practices, breath awareness (BA) and loving-kindness (LK), were selected because they are common to MBIs, understood in Buddhist traditions to produce divergent effects [2, 7], and have been shown in brief study contexts to produce stress relevant effects [18,19]. Gratitude (GT) was selected as a comparison contemplative practice because it is not a core MBI practice, but GT interventions have demonstrated benefits relevant to stress regulation [20]. Second, although GT shares similar content to LK (e.g., savoring the positive), LK is primarily oriented outward to others whereas GT is based on positive experiences with exogenous causes and is therefore primarily oriented inward to self [20]. Because stress enhances self-focus, the other versus self-orientations of LK and GT might interact with a stressor to different effect. As an attention control condition (CT), we modified an activity previously used as a control in gratitude research [21] so that it engaged similar levels of cognitive load as the other contemplative techniques, but without instructions for working with attention and distraction (as compared to BA), affect (as compared to LK & GT), or prosocial disposition (as compared LK & GT).

To interrogate the effects of these practices in the context of a stressor, we induced an acute stress experience, described as similar in magnitude to regular day-to-day stressors [11], by subjecting participants to a social-evaluative variant of the Cold pressor test (CPT) [2]. The present version of the CPT involved submerging the non-dominant hand in an ice-cold (~34 degree) bath of water for 3 minutes, while a member of the research team dressed in a white laboratory coat observed with an expressionless face. The CPT reliably elicits a subjective and autonomic stress response similar to other social stressors) [11, 22]. Adding the social-evaluative component has been found to stimulate Hypothalamus-Pituitary-Adrenal (HPA) axis activation as well (i.e., cortisol secretion) [22]. The reliable induction of a subjective and physiological stress response makes the paradigm well suited for disentangling potential differences in the ways contemplative practices impact stress regulation.

Based on prior research, we predicted that assignment to LK and GT would enhance positive affect, but all contemplative trainings would reduce negative affect relative to CT [8, 9, 23]. Second, we hypothesized that participants in the three contemplative conditions would demonstrate less reactivity to the stressor. We theorized that enhanced positive affect as a result of LK and GT practice would buffer the deleterious effects of the CPT, while BA practice would provide an attentional strategy that could be deployed to the same end. We operationalized less reactivity as 1) greater CPT completion rates, 2) lower reported CPT aversiveness, and 3) tempered increases in negative affect resulting from the CPT. Because enhanced cortisol production after a stressor has been shown to negatively bias appraisals of emotionally ambiguous faces [24], we predicted that LK and GT participants would rate novel neutral faces as more likable post-CPT. Finally, we hypothesized that the predicted buffering effect of LK and GT practice on CPT reactivity would promote higher levels of time donation.

## Materials and methods

The University of Wisconsin Madison's Education and Social Behavioral Sciences Institutional Review Board approved this study (IRB 2014–0572, Effects of brief practices). The study was preregistered on ClinicalTrials.gov (NCT02214264). Before the study began, all participants provided written informed consent. Written consent documents notified participants that the study was examining the impact of brief mental trainings and involved submerging the non-dominant arm in an ice water bath for 3 minutes.

### Participants

One hundred and sixty-six (*n* = 101 females, 60.84%) undergraduate students from a large, Midwestern university were recruited into the study for general psychology course credit. Undergraduates entered into an online study portal where several dozen research studies were open for participation. To avoid selection bias, the only information about the studies provided in the study portal are the duration of the study, the number of credits awarded following participation, and the number of study visits required. Participants that reported being pregnant, non-native English speakers, or under the age of 18 were excluded prior to signing up for the study through a prescreening process conducted on the online recruitment system (numbers of excluded participants unavailable). Four participants (2.4%) that arrived for the study session were excluded prior to study onset for not meeting inclusion criteria (English was not the primary language). A total of six additional subjects (3.6%; 4 female, 1 male, 1 no gender reported) were excluded from analyses due to dizziness following the CPT (*n* = 5) and experimenter error (*n* = 1).

Eighty-three percent of the final sample self-identified as White, 5.8% as Asian, 3.8% as Black or African American, 5.8% as more than one race, and 1.3% as unknown or did not report race. The mean age was 19.29 years old (*SD* = 0.74). The majority of participants had no prior meditation experience (*n* = 91, 58.33%). Fifty-five participants (33.3%) reported practicing some form of meditation once a week or more. Chi-square tests revealed no between-group differences in daily, weekly or last year meditation practice (χ^2^ (155) = 1.97, *p* = .579, χ^2^ (155) = 2.94, *p* = .401, χ^2^ (155) = 4.43, *p* = .218, respectively).

### Interventions

All four brief practices consisted of approximately 12 minutes of guided audio instruction provided by a female voice. The length of practice was determined in part by the existing brief practice literature, in which practices are often between 10–20 minutes in length [8, 16, 17] and by the fact that many popular contemplative applications involve practices of roughly this duration. Because the delivery of a practice might impact its effect and gold standard versions of these brief practices do not yet exist, contemplative practice selection was informed by the authority providing the practice. For BA and LK, we selected guided audio practices by a well-known American mindfulness and meditation teacher [25] whose personal training in these contemplative techniques afford her authority status. For GT, we based the practice off of two empirically validated protocols, one considered the “classic” gratitude intervention and the other found effective in inducing positive affect after 5-minutes of contemplation [21]. The attention control condition is a modified active control used in research on gratitude [21].

Structure and duration of the practices were matched as closely as possible. Concluding remarks were identical and included the suggestion that one could bring the practice or the feeling elicited into a variety of life experiences. Full transcripts are available in S1 Table.

#### Breath awareness

Breath awareness is a foundational mindfulness practice that involves maintaining attention on the physical sensations of the breath and gently bringing attention back to the breath when the mind has wandered. Emphasis was placed on allowing thoughts and other potential distractions to naturally come and go while continuing to return attention to the sensation of the movement of the breath [25]. See Supporting Information 1 for transcript.

#### Loving-kindness meditation

The classical intention of LK is to reduce self-focus and anger by developing feelings of warmth and kindness towards one’s self and others [26]. Participants began by repeating loving-kindness phrases such as “May I be safe”, “May I be happy” to themselves, then to someone who has helped them, followed by a person that is experiencing hurt, concluding with a neutral person. Similar to the breath awareness practice instructions, participants were told to allow thoughts and other potential distractions to come and go naturally while returning attention to the loving-kindness phrases and the object of the phrases [25]. See Supporting Information 1 for transcript.

#### Gratitude

The gratitude practice involved recalling and generating feelings of thankfulness. For the first five minutes, participants curated a list of events and people for which they felt grateful [21]. They next recalled a situation in which someone acted kindly toward them and were instructed to fully immerse in recollecting the experience, particularly paying attention to the feelings of thankfulness and gratitude that naturally emerge when reflecting on the kindness inherent in this experience. This was followed by a period of reflecting on and immersing oneself in all lived experiences that generate a feeling of gratitude. Throughout, instructions included referring back to the curated list. See Supporting Information 1 for transcript.

#### Attention control condition

Similar to the gratitude training, for the first five minutes, participants were instructed to visualize and describe (in writing) with as much detail as possible moving from the entrance of their home to their bedroom or, if they desired, throughout the entire home. Participants were then instructed to choose their favorite room and visualize its contents in detail (e.g., wall colors, art, windows, lighting). They were next instructed to notice the feeling associated with recollecting their favorite room. For the final minutes, participants could move on to a second room or continue experiencing their favorite room. This condition was designed to engage comparable levels of effortful attention as the contemplative trainings, without including techniques for working with distraction or generating positive feelings.

### Materials

#### Anxiety sensitivity index

The ASI [27] is a 16 item self-report of anxiety sensitivity, or the belief that anxiety experiences have negative results. Items including “When my stomach is upset, I fear that I might be seriously ill” are rated on a Likert-like scale from 0 (very little) to 5 (very much). Higher scores reflect greater anxiety sensitivity (α = .90). The ASI was included as a baseline measure so that any individual differences in anxiety sensitivity (i.e., nuisance variable) that might enhance the negativity of the CPT could be controlled for.

#### Gratitude questionnaire

The GQ-6 [28] is a six-item self-report assessing trait gratitude. Participants rate themselves on a scale from 1 (strongly disagree) to 7 (strongly agree) on items like “I am grateful to a wide variety of people.” Higher scores represent greater gratitude (α = .78). The GQ6 was included so that individual differences could be controlled for.

#### Implicit positive and negative affect task

The IPANAT [29] measures implicit affect by asking participants to rate the extent to which words from an artificial language express certain moods. Participants rate on a scale ranging from 0 (doesn’t fit at all) to 4 (fits very well) how well six words (e.g., SAFME) are described by 3 positive (e.g., happy) and 3 negative (e.g., tense) adjectives. Implicit positive and negative affect are calculated by averaging across the ratings on positive (α = .82) and negative adjective (α = .82). Higher scores represent greater implicit affect.

#### Positive and negative affect schedule

The PANAS Now [30] is a 20-item self-report of positive and negative affect in the moment. On a 1 (very slightly/not at all) to 5 (extremely) scale, participants rate their current state of positive (α = .95; e.g., interested, proud) and negative (α = .85; e.g., guilty, afraid) affect. Higher scores reflect more of the respective form of affect.

#### Cold pressor test

The Cold pressor test (CPT) is a widely used standardized physical stressor shown to reliably elicit autonomic stress reactivity [11, 12, 31]. Participants are asked to keep their non-dominant hand up to the elbow in an ice bath (~34 degree water) for three minutes while being observed at a close distance by an unexpressive experimenter wearing a white laboratory coat recording time.

The apparatus was constructed from an Igloo rigid 48-quart cooler. An NSF approved Taylor precision pocket thermometer was placed in the ice-water to provide real-time water temperature monitoring. A Styrofoam board that descended approximately half the depth of the cooler was inserted to subdivide the cooler, leaving a large area for participant arm submersion and a small area for an Aquatop 22W 317 GOH flow rate water-pump to maintain constant water flow and balance water temperature throughout the cooler. If the water temperature began to warm, the experimenter added a plastic bag containing ice (prefilled) to maintain a consistent water temperature of about 34 degrees. If the participant removed their arm at any point, the time of removal (in seconds from submersion) was recorded and the CPT was terminated.

#### Cold pressor test aversiveness rating

A three-item scale based on prior research [11, 32] was used to assess the subjective painfulness, unpleasantness, and stressfulness of the CPT. Participants rated the questions “How painful was the task?”, “How unpleasant was the task?”, and “How stressful was the task?” on a 0 (not at all) to 100 (very) visual slider. Based on prior results showing a stronger relationship between the painfulness and unpleasantness questions [22] and previous implementation of only the pain and unpleasantness items [32], we aggregated these two items into an “aversiveness” scale (α = .89). Here, we define aversiveness as the degree to which the CPT was experienced as painful and unpleasant.

#### Perceptions of brief practices questions

After the CPT, we asked participants to rate how much attention and effort they felt they allocated to the practice, how likely they felt they were to engage in the practice again, and how well they felt the practice helped them cope with the CPT. The attention question was rated on a visual slider anchored at 0 (None at all) and 100 (To the best of my ability). The use again and cope questions were anchored on a visual slider at 0 (Not at all) and 100 (Very).

#### Face ratings

Prior research has demonstrated that ratings of novel neutral as well as emotionally ambiguous faces are influenced by emotional state, by priming with affective images, and by dispositional characteristics in a phenomenon described as affective bias or coloring [33, 34]. Here, novel neutral African American, White, and Asian female and male faces appeared on a computer screen with a visual slider underneath ranging from 0 (not at all likable) to 100 (very likable). Faces were drawn from the XM2VTSDB multi-modal face database [35], the NimStim database [36], and the Montreal Set of Facial Displays of Emotion [37]. Faces were cropped just above the hair and below the chin, converted to black and white, and edited (e.g. to remove distinctive facial hair and eyeglasses). Participants rated 60 faces as quickly as possible based on their initial feeling upon seeing the face. Faces appeared in semi-random order such that roughly equivalent numbers of male and female faces for each race were rated at baseline and after the CPT. Test-retest reliability in this sample was high, *r*(155) = .856, *p* < .001.

#### Time donation

After notifying participants that they had completed the study and were free to go, the experimenter asked if they would be willing to provide ratings on a new face set the researchers were normalizing. We used as a dependent variable the number of participants that donated time (0 = did not donate, 1 = donated).

### Procedure

Participants were recruited through an online study recruitment portal for introductory level psychology courses. After participants signed-up, they were met at the laboratory by trained undergraduate research assistants and consented. We used a random number generator to generate a random order sequence for the numbers 1, 2, 3, and 4 (corresponding to each study condition). Participants were allocated to study condition based on this sequence and their testing time (i.e., simple randomization). Experimenters were kept blind to study hypotheses, the content of the conditions (i.e., they did not know that BA, LK, GT, and CT were the practices), and the key associating condition number with practice type. During the practice portion of the study, the participant put on headphones and the experimenter clicked on the audio file with the appropriate number (i.e., 1, 2, 3, or 4) and then left the room as the participant practiced. Instructions never explicitly named the practice and the word mindfulness was never used to avoid demand characteristics. Thirty-four participants were assigned to BA (58.82% female), 42 to LK (71.42% female), 41 to GT (53.66% female), and 39 to CT (64.10% female).

**Fig 1:**
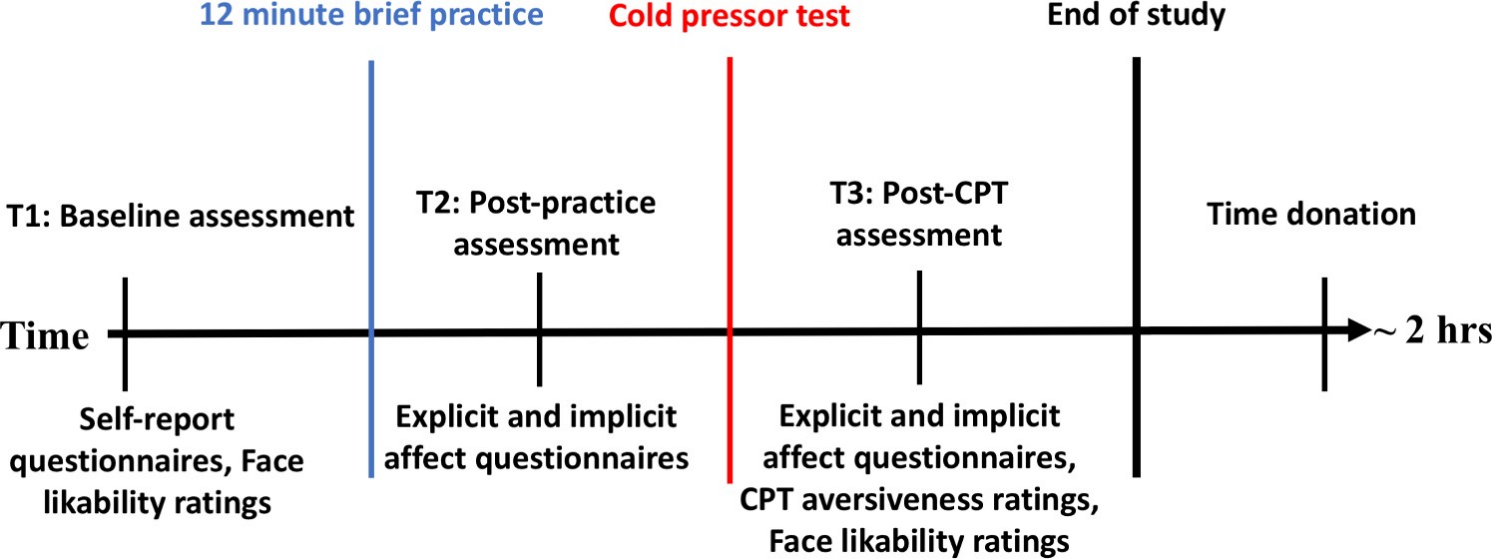
Study design.

**Fig 2:**
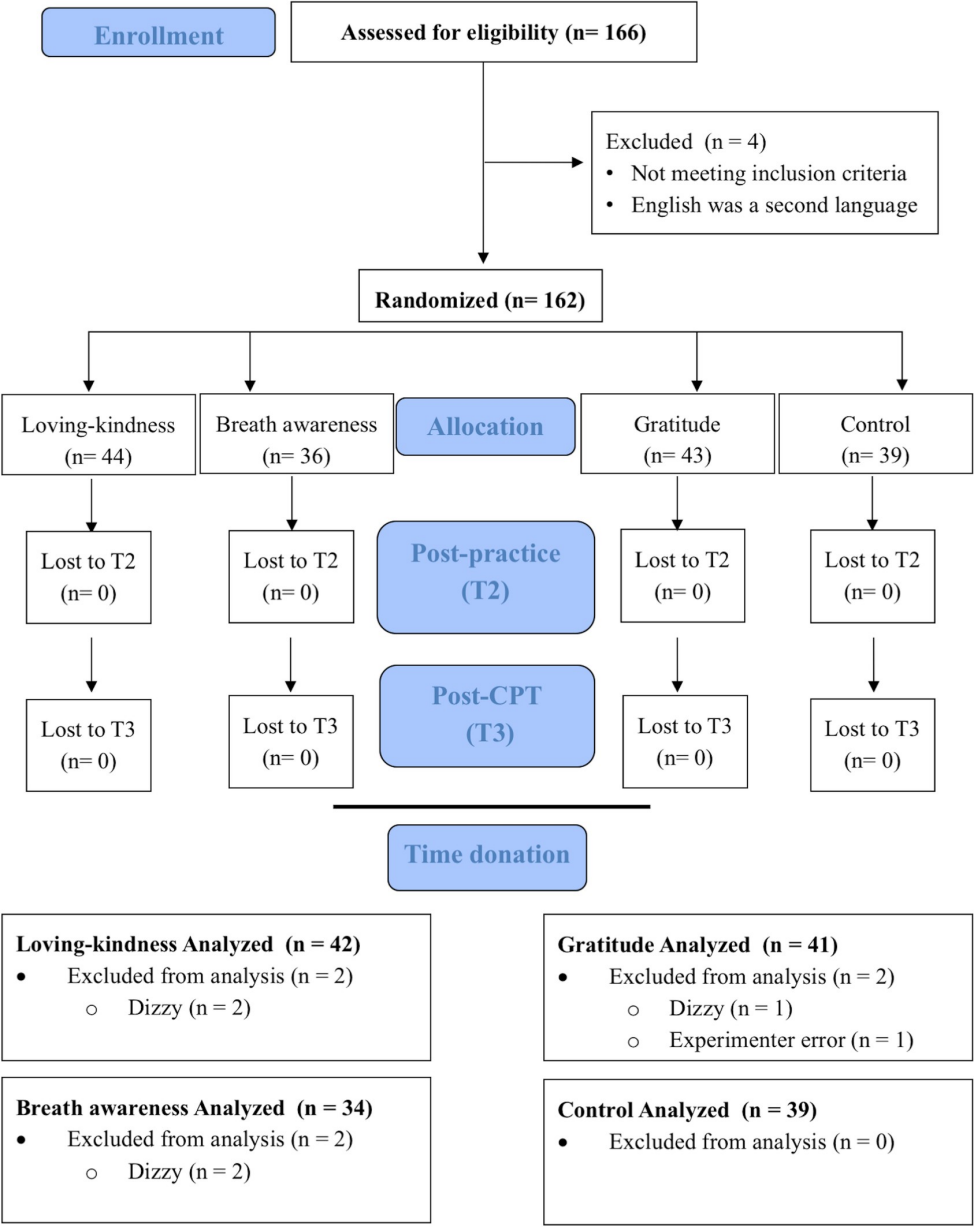
CONSORT diagram.

Participants first completed all measures except CPT aversiveness rating and the time donation task on a desktop computer. Qualtrics [38] was used for all self-report assessments and EPrime psychological testing software [39] was used for behavioral tasks. Behavioral measures of working memory (i.e., OSPAN) [40] and self-regulation (i.e., Hand-grip task) [41] were used but are not included in the present analyses [42]. Participants then completed their assigned training. After training, affect (implicit and explicit) was again rated (i.e., T2). The CPT was administered followed by a third assessment of affect, a second Face rating task, and CPT aversiveness ratings (i.e., T3). Once complete, research staff informed the participant that the experiment was over, providing an unobtrusive and ecologically valid opportunity to donate time by asking whether participants were willing to stay longer to help norm a set of novel face images (See Fig 1, Fig 2). The testing paradigm took approximately two-hours. All procedures were approved by the university’s Institutional Review Board.

### Analytical approach

We used multiple regression models with GROUP as a categorical predictor to examine the impact of practice type on outcome change. Before correction for multiple comparisons, significantly between-group baseline differences were observed on the GQ6, IPANAT negative subscale, anxiety sensitivity (ASI), and face ratings. We therefore included all four as centered covariates (to aid interpretation). No outliers were observed and models met assumptions for Ordinary Least Squares (OLS) modeling, except where noted below.

Exploratory analyses (i.e., *post-hoc*) were controlled for at a false discovery rate of α = .05 by employing False Discovery Rate correction (FDR) [43]. We report effect sizes (Cohen’s *d* or odds ratio, where appropriate) in addition to statistical significance as a meaningful indicator of group difference [44]. Significance level was set as *p <* .05. All tests were two-tailed. All data processing and analysis was done using *R* Studio v.1.1.414 [45]. The generic model for most analyses can be written as:

$$Y_{change}=\alpha +\beta_{1}(GROUP)+\beta_{2}({ASI}_{centered})+\beta_{3}({IPANAT\;Negative}_{centered})+\beta_{4}({Face\;ratings}_{centered})+\beta_{5}({GQ6}_{centered})+\varepsilon$$

where Y_change_ is the raw change in a given outcome (e.g., Time2 –Time1 implicit positive affect), GROUP is a dummy coded categorical variable for practice condition, ASI, IPANAT negative, Face ratings, and GQ6 are centered covariates and ε is residual error.

#### Power analyses

The study was designed to test for group effects as well as moderation of group effects by individual differences. For the latter design, *a priori* power analyses conducted with GPower 3.1 [46] revealed small magnitude (i.e., Cohen’s *d* = 0.30) moderation effects could be detected with .80 power with a sample of *N* = 350 participants. We thus planned to recruit 350 participants. An unanticipated problem that arose during the study period reduced the study duration by about 30%, leading to a final sample of *N* = 156.

## Results

Descriptive statistics for all continuous measures broken down by group and measurement time point can be found in Table 1.

**Table 1:** Means and standard deviations (*SD)* for continuous variables at each measurement point.

	Loving-kindness (*n* = 42) Female *n* = 30			Breath Awareness (*n* = 34) Female *n* = 20			Gratitude (*n* = 41) Female *n* = 22			Control (*n* = 39) Female *n* = 25			
	T1	T2	T3	T1	T2	T3	T1	T2	T3	T1	T2	T3	T1 differences
**PANAS positive**	2.8 (0.71)	2.75 (0.77)	2.48 (0.82)	2.92 (0.72)	2.69 (0.92)	2.44 (0.99)	2.63 (0.61)	2.8 (0.88)	2.59 (0.88)	2.65 (0.73)	2.61 (0.82)	2.56 (0.82)	*p*s > .05
**PANAS negative**	1.26 (0.23)	1.17 (0.30)	1.63 (0.60)	1.36 (0.33)	1.21 (0.33)	1.68 (0.51)	1.29 (0.32)	1.18 (0.26)	1.90 (0.71)	1.37 (0.37)	1.22 (0.34)	1.59 (0.55)	*p*s > .05
**IPANAT positive**	2.11 (0.41)	2.23 (0.51)	1.96 (0.49)	2.12 (0.38)	2.23 (0.52)	2.09 (0.53)	2.03 (0.46)	2.15 (0.51)	1.87 (0.60)	2.09 (0.43)	2.08 (0.47)	1.93 (0.46)	*p*s > .05
**IPANAT negative**	1.91 (0.37)	1.73 (0.42)	1.98 (0.52)	2.01 (0.44)	1.89 (0.47)	2.03 (0.52)	1.73 (0.38)	1.57 (0.38)	1.83 (0.58)	1.83 (0.36)	1.79 (0.36)	1.9 (0.51)	BA > GT** BA > CT*
**Face ratings**	52.04 (8.66)	_	50.10 (9.09)	50.41 (8.56)	_	49.22 (8.37)	46.76 (11.26)	_	44.22 (12.90)	46.60 (13.04)	_	44.63 (13.82)	LK > GT*
**CPT**	_	_	83.41 (16.34)	_	_	79.05 (17.01)	_	_	87.03 (15.89)	_	_	78.65 (18.70)	N/A
**ASI**	2.43 (0.70)	_	_	2.36 (0.70)	_	_	2.07 (0.58)	_	_	2.16 (0.49)	_	_	LK > GT** BA > GT*
**GQ6**	6.52 (0.54)	_	_	6.35 (0.71)	_	_	6.37 (0.66)	_	_	6.06 (1.23)	_	_	LK > CT *

Notes: PANAS = Positive and Negative Affect Schedule; positive and negative subscales. IPANAT = Implicit Positive and Negative Affect Test; positive and negative subscales. CPT = Cold pressor test aversiveness rating. ASI = Anxiety Sensitivity Index. GQ6 = Gratitude 6 Questionnaire. T1 = Baseline assessment before training. T2 = Assessment immediately following training. T3 = Assessment immediately following the Cold pressor test.

*** = *p* < .001

** = p < .01

* = p < .05.

Baseline between-group contrasts were not controlled for multiple comparisons. After controlling for multiple comparison with False Discovery Rate, GT and BA differ at baseline on IPANAT Negative and GT and LK differ at baseline on ASI. There are no other between-group baseline differences. Loving-kindness = LK; Breath awareness = BA; Gratitude = GT; Control = CT.

### Training effects on affect (Δ_T2 –T1_)

**Fig 3:**
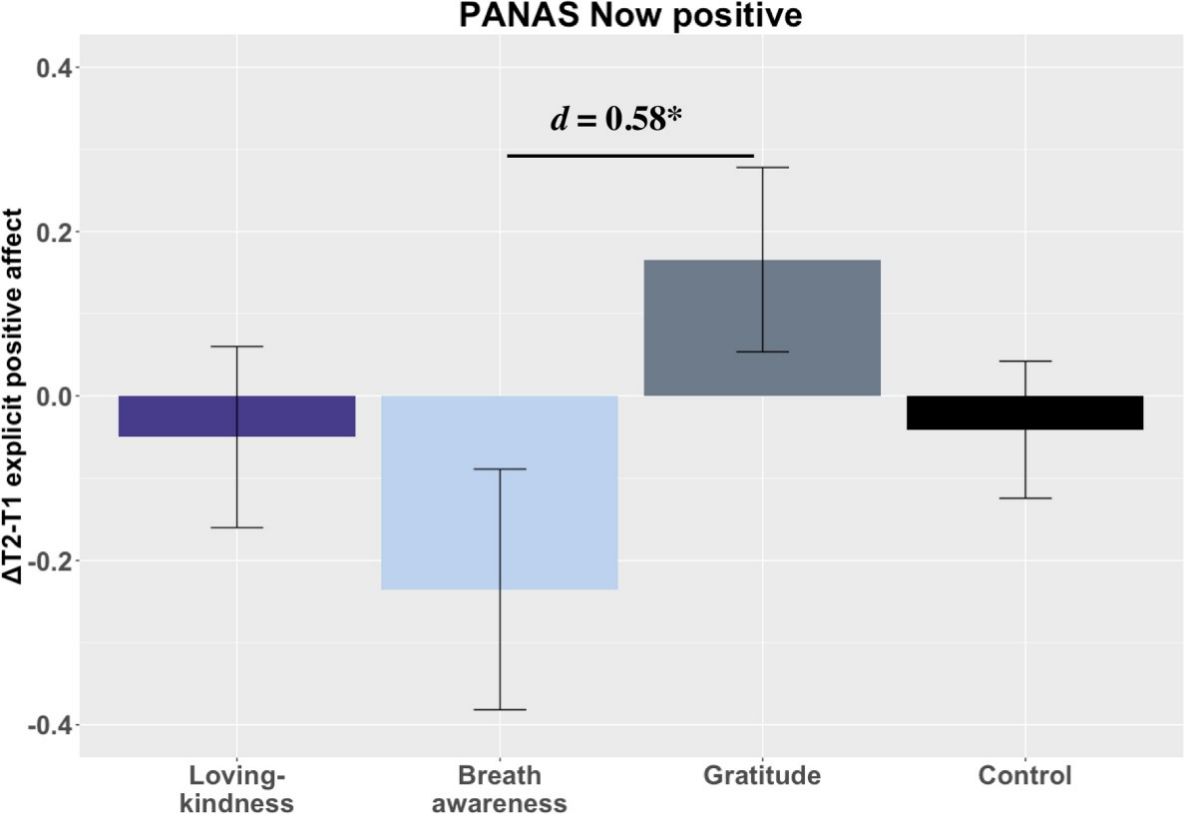
Training changes in positive affect. Notes: *** = *p* < .001, ** = p < .01, * = p < .05, *t = p* < .10. Loving-kindness = LK; Breath awareness = BA; Gratitude = GT; Control = CT.

We found limited support for the hypothesis that LK and GT would increase positive affect compared to CT (Fig 3A and 3B). In fact, only GT showed gains in explicit positive affect (**Δ** *=* 0.17, *SD* = .72) with LK (and BA) showing average slight decreases post-training (**Δ** *=* -0.05, *SD* = .71 and **Δ** *=* -0.24, *SD* = .85, respectively). Neither LK nor GT change differed from CT (*p*s > .05), but in a *post-hoc* test, GT showed moderate magnitude, significant gains compared to BA t(69) = 2.51, *p* = .039, *d* = 0.58 95% CI[0.11, 1.05] (corrected). On implicit positive affect, LK and GT had small magnitude, but non-significant gains compared to CT t(75) = 1.46, *p* = .147, *d* = 0.32 CI[-0.12, 0.77] and t(74) = 1.80, *p* = .073, *d* = 0.40 CI[-0.05, 0.85], respectively.

**Fig 4:**
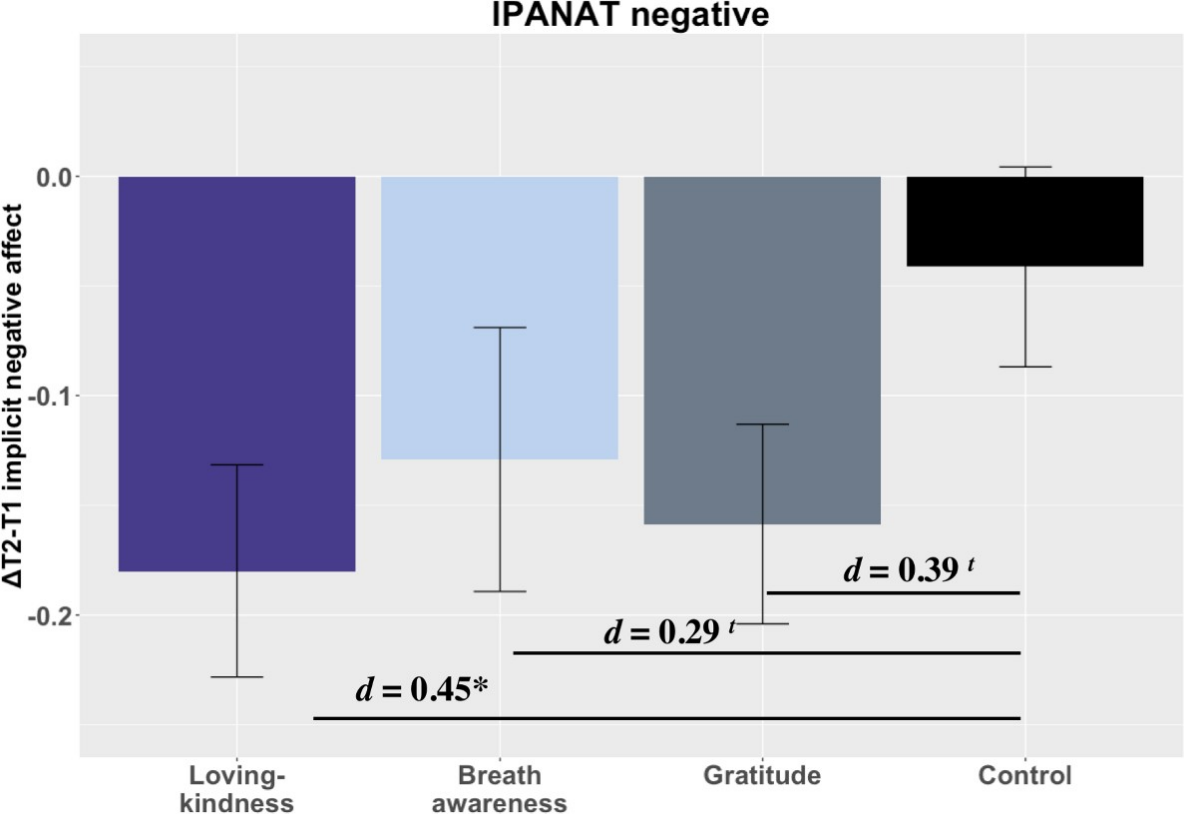
Training changes in negative affect. Notes: *** = *p* < .001, ** = p < .01, * = p < .05, *t = p* < .10. Loving-kindness = LK; Breath awareness = BA; Gratitude = GT; Control = CT.

There was no evidence for contemplative training effects on explicit negative affect compared to CT (all *p*s > .05; Fig 4A). As predicted, all contemplative groups demonstrated greater reductions in implicit negative affect than CT, with LK showing moderate magnitude significantly greater reductions t(76) = 2.33, *p* = .021, *d* = 0.45 CI[0.06, 0.84] and BA and GT showing small magnitude, non-significant reductions, t(68) = 1.45, *p* = .149, *d* = 0.29 CI[-0.5, 0.73] and t(75) = 1.77, *p* = .079, *d* = 0.34 CI[-0.11, 0.70], respectively (Fig 4B).

### Intervention effects on stressor experience

We hypothesized that the three contemplative conditions (i.e., BA, LK, & GT) would promote more effective regulation of the stressor. Consistent with this prediction, there was a moderate magnitude non-significant difference in completion rates such that the contemplative conditions (BA, LK, & GT) were 3.08 times more likely to complete the CPT than CT, OR = 3.08 95% CI[1.03, 9.15], *p* = .054. Participants in BA and LK (i.e., common MBI practices) were 4.43 times more likely to complete the CPT than participants in non-MBI trainings (GT & CT), OR = 4.43 CI[1.18, 16.12], *p* = .054 (corrected).

**Fig 5:**
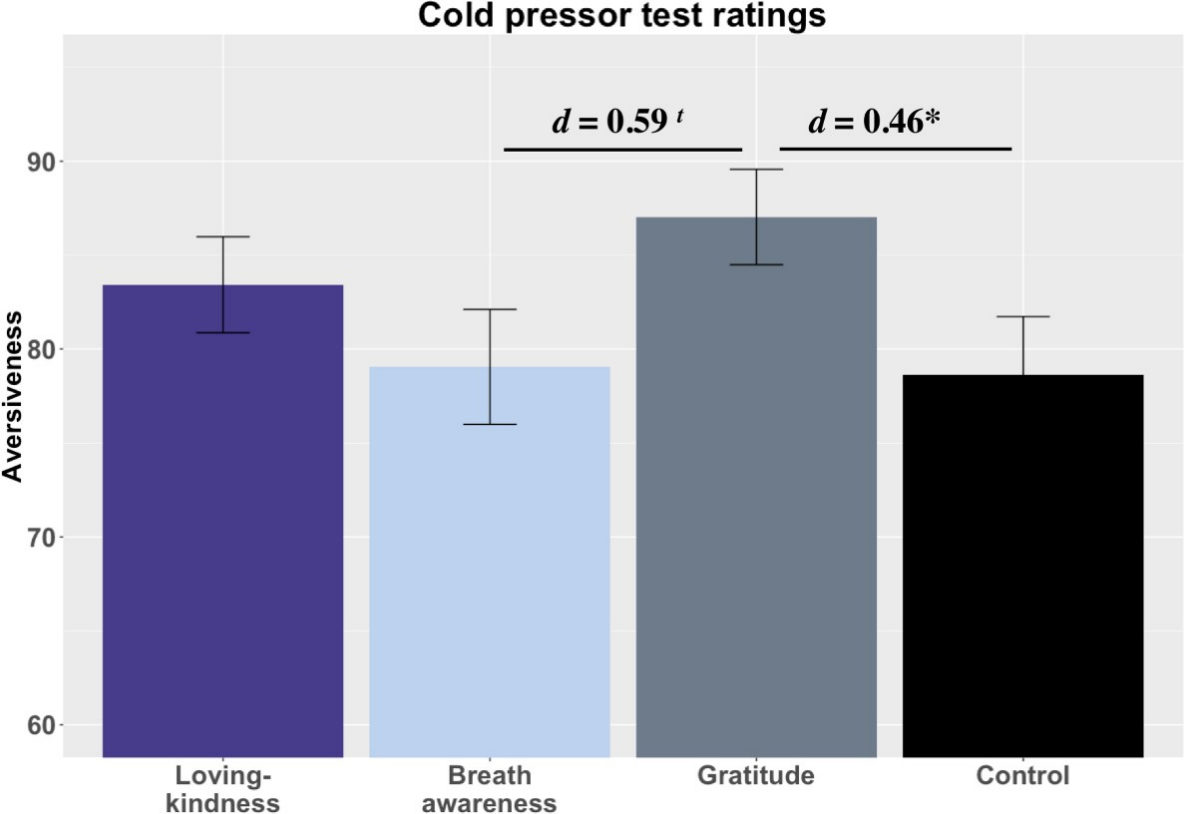
Cold pressor aversiveness ratings. Notes: *** = *p* < .001, ** = p < .01, * = p < .05, *t = p* < .10; 4A. Loving-kindness = LK; Breath awareness = BA; Gratitude = GT; Control = CT.

We next examined group differences in CPT aversiveness ratings (Fig 5). Because some participants removed their hand before three minutes had elapsed, we included total duration (in seconds) of hand immersion as a covariate. In addition, model results deviated from several assumptions of OLS regression (i.e., heteroscedasticity, normality of residuals). To ensure results were not impacted by these deviations, we employed Huber’s M-estimation robust regression to down-weight more extreme points.

LK and BA did not differ from CT in aversiveness ratings (*p*s > .30), but contrary to predictions, GT rated the experience as moderately and significantly more aversive than CT, t(69) = 2.00, *p* = .048, *d* = 0.46 CI[0.00, 0.92]. In a *post-hoc* test, we found that GT also rated the experience as moderately but after correction not significantly more aversive than BA t(63) = 2.46, *p* = .06, *d* = 0.59 CI[0.01, 1.08] and slightly more aversive than LK t(73) = 1.26, *p* = .277, *d* = 0.28 CI[-0.16, 0.73] (corrected). Trait gratitude explained significant unique variance in CPT aversiveness, but unexpectedly, higher scores predicted greater aversiveness, *b* = 4.61, *SE* = 1.72, *p* = .008. Baseline anxiety sensitivity also predicted unique variance in aversiveness, with higher scores predicting greater aversiveness *b* = 7.07, *SE* = 2.19, *p* = .002.

### Change in affect after stressor (ΔAffect _T3—T2_)

**Fig 6:**
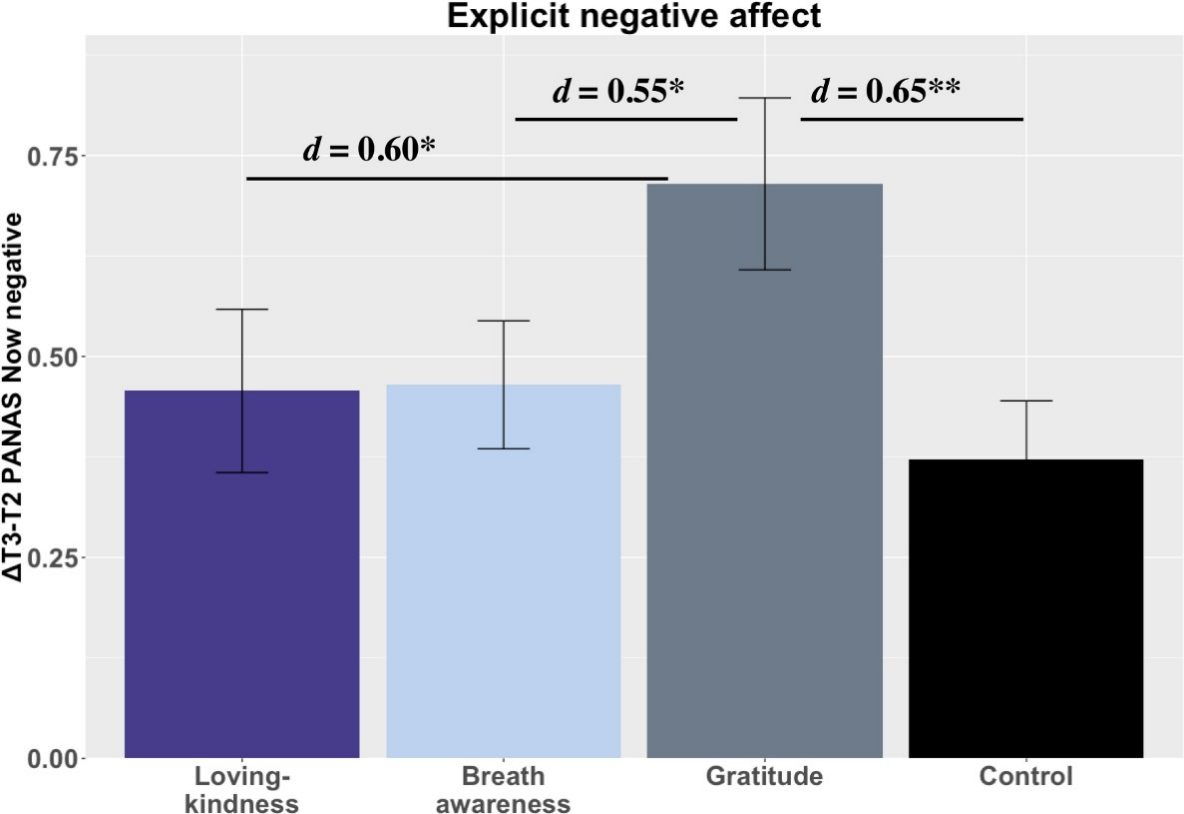
Cold pressor effects on negative affect. Notes: *** = *p* < .001, ** = p < .01, * = p < .05, *t = p* < .10; 4A. Loving-kindness = LK; Breath awareness = BA; Gratitude = GT; Control = CT.

As expected, the CPT increased explicit negative affect in all groups (see Fig 6). Only GT significantly differed from CT, but opposite the predicted direction. GT reported greater gains in explicit negative affect t(74) = 2.86 *p* = .005, *d =* 0.65 CI[0.18, 1.08]. In *post-hoc* tests, GT increases in explicit negative affect were significantly greater than both LK and BA as well, t(75) = 2.64, *p* = .021, *d* = 0.60 CI[0.14, 1.07] and t(69) = 2.52, *p* = .021, *d =* 0.55 CI[0.11, 0.99], respectively (corrected). There were no differences between contemplative practice conditions and CT in CPT related changes in implicit negative affect (all *p*s > .05).

### Cold pressor effects on face likability ratings

We found no evidence for our hypothesis that LK and GT would promote higher likability ratings than CT following the CPT (*p*s > .05).

### Post-hoc interaction analysis between practice effects and stressor

**Fig 7:**
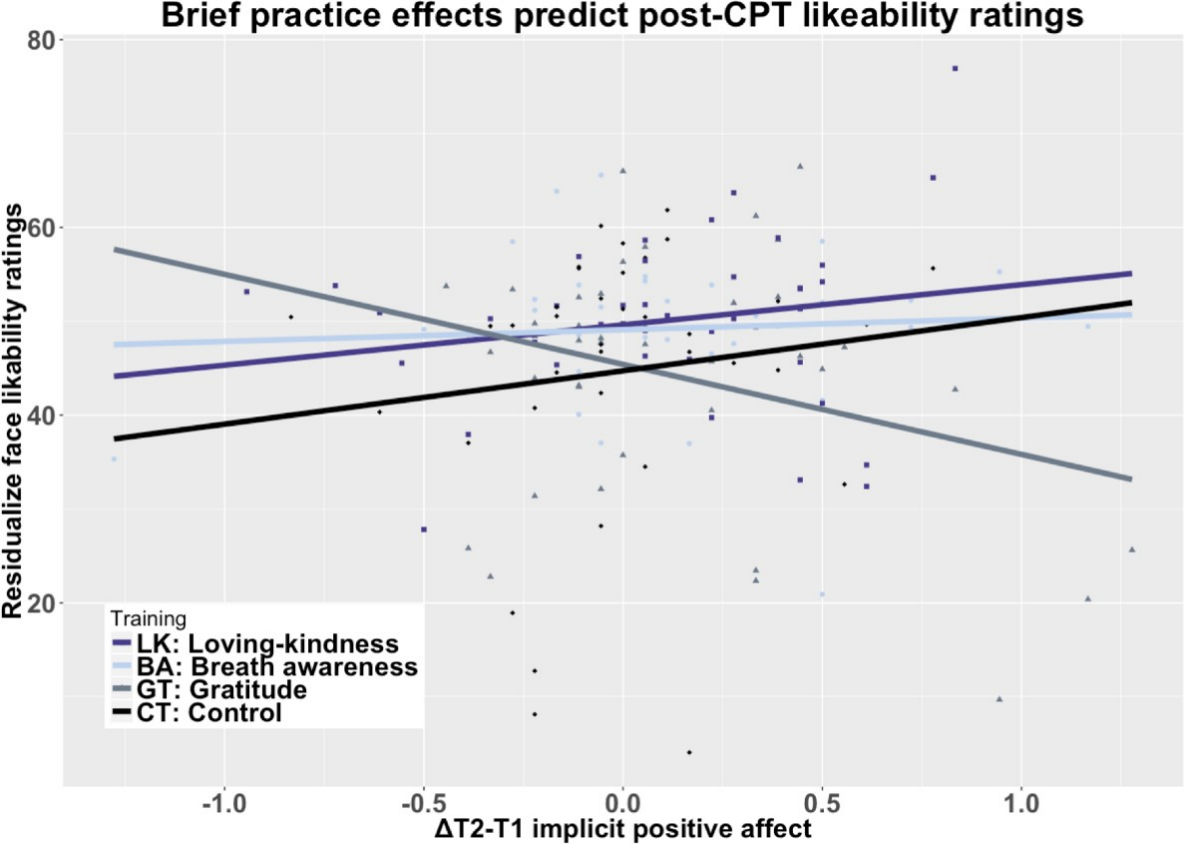
Practice induced positive affect x GROUP interaction predicts stressor-related negative affect. Notes: Explicit positive affect following practice in GT predicts stressor-induced increases in negative affect whereas in BA practice-related explicit positive affect gains are protective against stressor-induced negative affect t(68) = 3.50, *p <* .002, *d* = 0.81 CI[.33, 1.29] (corrected).

Because we found higher reactivity to the stressor in GT despite practice gains in positive affect (i.e., **Δ**T2—T1), we ran exploratory multiple regressions examining whether gains in positive affect interacted with GROUP to predict differences in CPT aversiveness, post-CPT changes in negative affect, and post-CPT face likability ratings. Brief practice gains in explicit positive affect did not interact with GROUP to significantly predict aversiveness or face likability ratings, but explicit positive affect gains in GT following the brief practice predicted significantly greater CPT-related increases in implicit negative affect compared to BA t(68) = 3.50, *p <* .002, *d* = 0.81 CI[.33, 1.29] (corrected, Fig 7).

**Fig 8:**
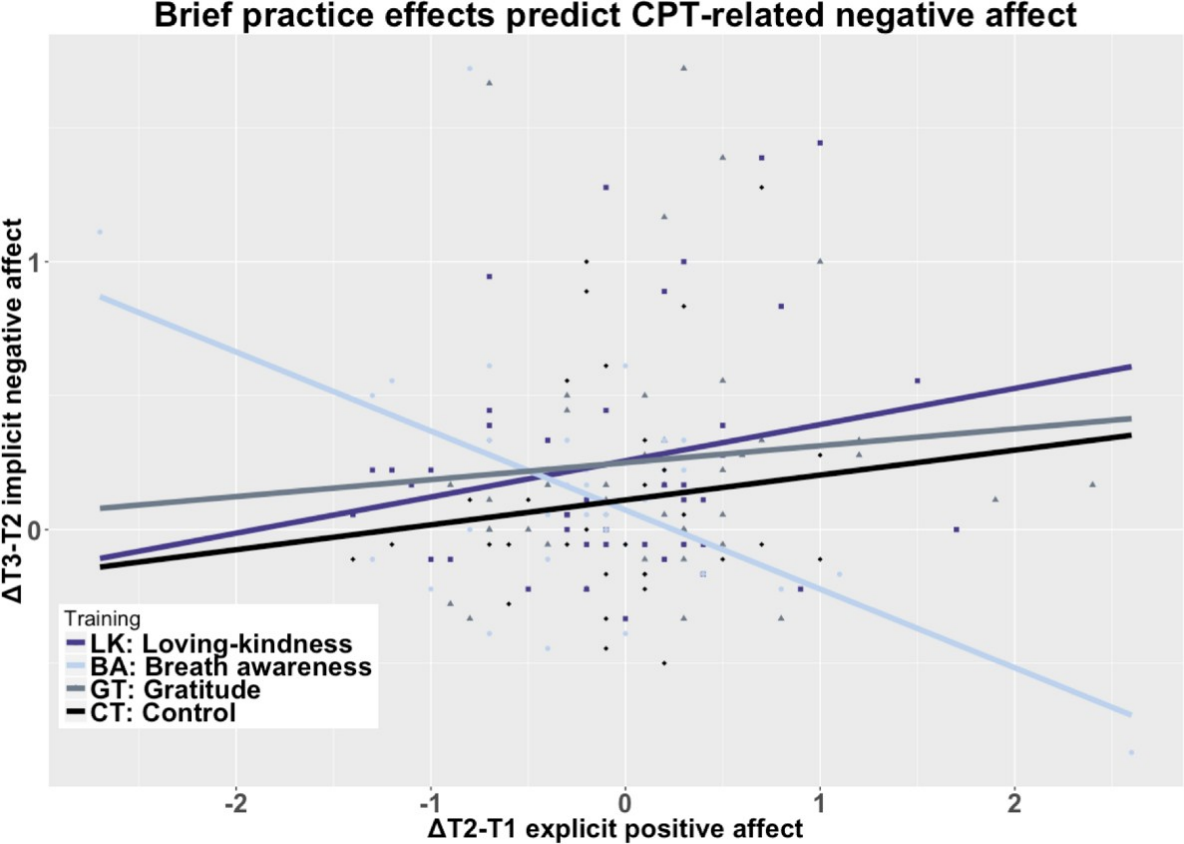
Positive affect training gains x GROUP predict post-CPT face likability ratings. Notes: Implicit positive affect following practice in GT predicts stressor-induced decreases in face likability ratings whereas in LK, BA, and CT practice-related implicit positive affect gains predict higher likability ratings GT-LK t(76) = -2.68, *p* = .020, *d* = -0.40 CI[0.1, 0.70], GT-BA t(68) = -2.51, *p* = .020, *d* = -0.39 CI[-0.71, -0.07], and GT-CT t(73) = -2.12, *p* = .036, *d* = -0.33 [-0.65, -0.01] (corrected).

Practice gains in implicit positive affect significantly interacted with GROUP to differentially predict post-CPT face likability ratings, but not changes in negative affect or CPT aversiveness. Specifically, in GT greater implicit positive affect training gains predicted significantly lower residualized post-stressor face likability ratings compared to LK t(76) = -2.68, *p* = .020, *d* = -0.40 CI[0.1, 0.70], BA t(68) = -2.51, *p* = .020, *d* = -0.39 CI[-0.71, -0.07], and CT t(73) = -2.12, *p* = .036, *d* = -0.33 [-0.65, -0.01] (corrected; Fig 8).

#### Perceptions of practice

Breath awareness reported the practice being significantly more helpful in coping with the CPT than CT t(74) = 2.51, *p* = .013, *d* = 0.59 CI[0.11, 1.07] and moderately but not significantly more helpful than GT t(69) = 1.91, *p* = .056, *d* = 0.44 CI[-0.02, 0.91]. There were no between-group differences in reports on the likelihood of future use or the level of attention allocated to the practice (all *p*s > .10).

### CPT effects on time donation by training

**Fig 9:**
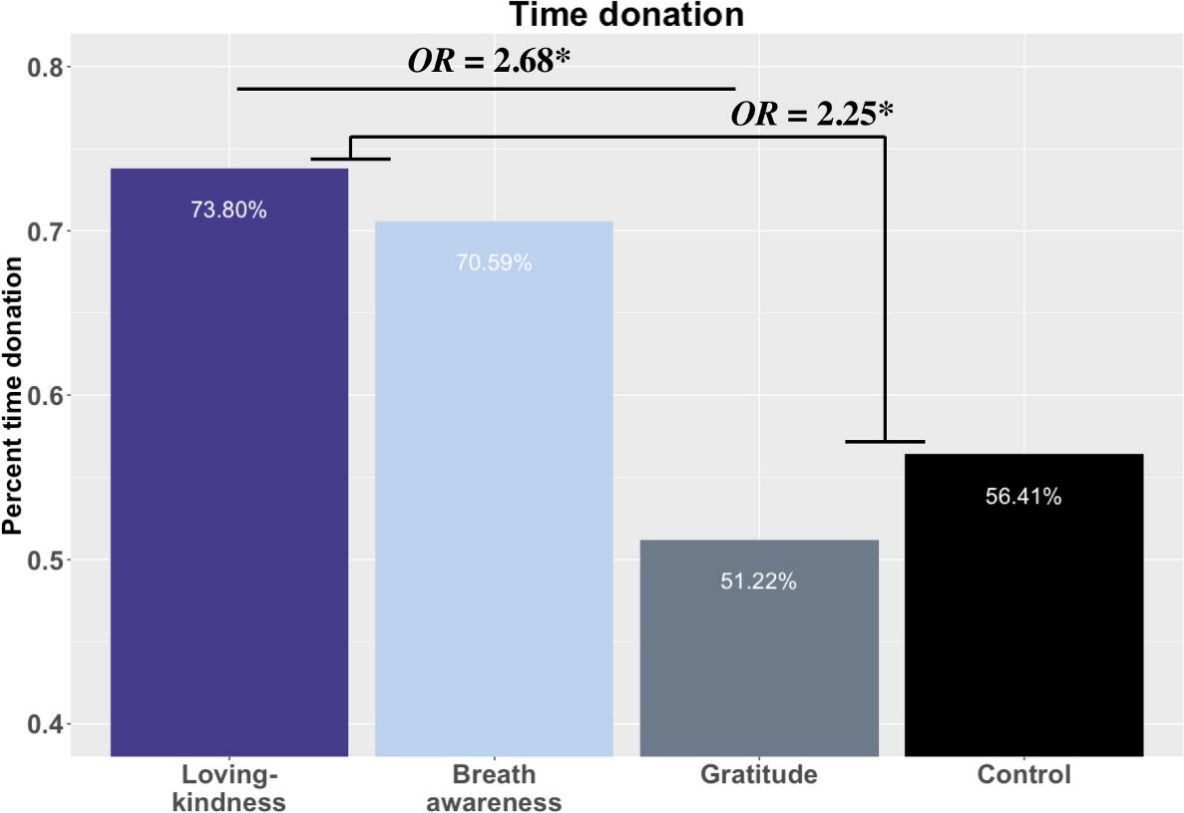
Training effects on generosity. *** = *p* < .001, ** = p < .01, * = p < .05, *t = p* < .10; 4A. Loving-kindness = LK; Breath awareness = BA; Gratitude = GT; Control = CT.

We found partial support for our hypothesis that LK and GT would donate more time. In line with our hypothesis, LK participants were the most likely to donate time (73.81%), but contrary to prediction, GT participants were the least likely (51.22%). Chi square tests revealed that LK participants were significantly more likely to donate time than GT, OR = 2.68 CI[1.07, 6.74], *p =* .050 (corrected). Breath awareness participants had the second highest donation rate (70.59%), followed by CT (56.41%). Participants in BA donated time 2.29 times more often than GT, but this difference was not significant OR = 2.29 CI[0.88, 5.96], *p =* .088 (corrected). Because LK and BA are common components of MBIs and the proportion of donation was descriptively different than observed in CT and GT, we combined LK and BA and compared the likelihood of time donation to GT and CT combined. A Fisher’s exact test revealed that together LK and BA donated time significantly more often than GT and CT, OR = 2.25 CI[1.16, 4.39], *p* = .048 (corrected, Fig 9).

## Discussion

In many traditional contemplative training regimens, practice techniques are understood to produce different and sometime unique effects. In the context of a brief practice and an acute stressor, the present experiment was designed to examine whether three forms of contemplative practice (Loving-kindness, breath awareness, and gratitude) differed in impacts on affect after training and on affect and behaviors after a stressor compared to an attention control condition. We observed subjective and behavioral divergences between contemplative practice conditions, most notably in response to the Cold pressor test. Gratitude appears to be particularly unsuitable as a buffer against a stressor, at least in novices experiencing a brief training. Participants in GT reported significantly higher CPT aversiveness than CT and moderately higher ratings than BA. In addition, GT demonstrated greater stressor-related increases in explicit negative affect relative to all other conditions. Assignment to GT also predicted a significantly lower likelihood of time donation post-stressor compared to LK, and although not significantly different, a moderate magnitude lower likelihood of time donation than BA.

Why gratitude, an intervention with evidence as a positive affect booster (partially replicated here), led to greater reactivity to the stressor is not clear. Above and beyond practice assignment, higher levels of trait gratitude predicted significantly greater CPT aversiveness. Interestingly, GT gains in positive affect following brief practice appear to have intensified rather than mitigated the deleterious influence of the CPT. Individuals in GT showing the greatest implicit positive affect gains had the lowest residualized likability ratings of novel neutral faces post-stressor, a significantly different pattern than observed in LK, BA, or CT. Moreover, greater practice-related gains in explicit positive affect in GT predicted larger stressor-related gains in explicit negative affect compared to BA. This pattern of results suggests that whatever boost in positive affect attended brief gratitude practice interacted with the stressor to magnify reactivity rather than buffer against it.

Loving-kindness and BA, different practice types often conflated under the generic banner of mindfulness practice, conferred relatively equivalent benefits. We observed no evidence for differential effects on affect as a consequence of practice. Nor did we find evidence that assignment to LK or BA produced differential responses to the CPT (e.g., no significant differences on CPT aversiveness ratings, post-CPT increases in negative affect, or time donation). In fact, participants in LK and BA were the most likely to donate time, and when combining these common constituents of MBIs and comparing time donation, LK and BA donated time at more than twice the rate of GT and CT. Although not significantly more likely after error control, LK and BA completed the CPT at 4.43 times the rate of GT and CT.

Given the prosocial nature of LK and GT, we predicted that assignment to these practices would predict greater time donation. As expected, LK practice led to the highest proportion of time donation, but GT practice led to the lowest proportion. The breath awareness group, not an inherently prosocial practice, produced the second highest proportion of time donation. Condon, Desbordes, Miller, and Desteno [47] found that 8-weeks of either mindfulness (i.e., no LK) or LK practice (i.e., no mindfulness) increased responses to others’ suffering more than wait-list control, with no differences between them, a finding consistent with these data.

Considering the pattern of findings in totality, one might conclude that BA and LK are similar practices that produce similar outcomes. Therefore, binning them together as mindfulness practices is warranted. We caution against such a conclusion for several reasons. First, BA and LK are on their face different practices. Micro-phenomenological interviews on similar practices confirm that subjective experiences of these practices are different [48] and longer-term practice in them has been shown to produce divergent outcomes [17]. Most important, our measures and those employed by Condon and colleagues [47] are coarse, evaluating observable behaviors but not the motivation behind the behavior. A compelling area for future research is the design of outcome measures that can differentiate not only whether one acts prosocially but also why.

Brief practice studies are valuable. They afford a view of practice effects in a controlled laboratory environment, eliminating variability inherent in exposure to contemplative practices outside of the lab. As growing numbers of the public come into contact with contemplative practices through applications on smartphones or programs on the web that are at least initially brief practice scenarios, these designs provide important knowledge about practice effects in this format (e.g., brief, guided audio). These data make clear that in the context of brief practice with novices, LK, BA, and GT diverge significantly on some outcomes, particularly after provocation with a stressor.

Although our findings indicate that implementing a brief gratitude practice to induce positive affect before a potentially stressful experience may be ill-conceived, it is important to avoid extrapolating brief practice study findings to longer-term contemplative training. Longer-term practice may cultivate or activate processes that are not elicited initially. For example, dispositional gratitude has been found to be protective longitudinally against stress and depression [49], and longer-term gratitude interventions show a number of benefits, including greater well-being, reduced negative affect, and increased connection with others [50]. In the context of an acute stressor, gratitude practice may enhance the negativity of the stressor, but in other contexts and as a result of continued practice, the weight of evidence supports gratitude as an important element of well-being [50].

### Limitations

This study has several limitations. The sample is sufficient to detect moderate magnitude effects or larger, making it likely that small and small-to-moderate magnitude effects of potential interest and importance went undetected. Second, there does not yet exist gold standard brief trainings of any contemplative practice. The potential variability in ostensibly the same practices’ instructions may meaningfully impact outcomes. Our practice selection criteria were designed to ensure that LK and BA were consistent with MBSR presentations of those practices and GT was as close to an evidence-supported GT training as possible, but the lack of consensus gold standard presentations remains a limitation. Relatedly, the brief trainings were not recorded by the same individual. All trainings were matched on the gender of the voice (female), structure, and duration, but it is possible that one voice was more compelling than another, affecting training response. Finally, the sample is relatively heterogenous in its racial/ethnic composition and comprises a very narrow age cohort. These results may not generalize across diverse cultures or age cohorts.

One benefit of the study design was temporal precision in affective change as a consequence of brief practice and then the stressor. We selected a state affect measure so that participants would report on their affect in the moment, rather than retrospectively, but recall bias might have influenced reporting. Finally, the attention control condition was based on a control used in prior research, but the evidence of its impacts is limited, making it unknown how well matched this control was to the three contemplative practice conditions.

### Conclusions

This study is among the first to provide proof of concept that even brief practice in different contemplative practice styles promotes divergent effects and that some practices may be better suited to supporting stress regulation than others. By comparing across several types of practices, these data add to a small but growing evidence base that even among practices often subsumed under a single designation (e.g., mindfulness or contemplative), it is important to consider the specific practices that comprise an intervention. A final strength of this study is the inclusion of self-report and behavioral outcome measures. By combining these and an unobtrusive, ecologically valid opportunity to donate time, this study provided a rich time course of training and stressor effects on affect and behavior.

## Supporting information

S1 Table Training scripts. (DOCX)
