# Supplementary material for: Divergent effects of brief contemplative practices in response to an acute stressor: A randomized controlled trial of brief breath awareness, loving-kindness, gratitude or an attention control practice
Source: PLoS One. 2018 Dec 12;13(12):e0207765. doi: 10.1371/journal.pone.0207765 (PMC6291192; doi:10.1371/journal.pone.0207765)
Supplement: S1 Table — (DOCX) [file pone.0207765.s001.docx]

**Loving Kindness (12 minutes 13 seconds)**

0.0 – 0.11: *Bell*

0.11 – To begin with, you can sit comfortably and relax. You don’t have to feel self-conscious as though you are about to do something special or weird. Just be at ease. It helps if your back can be straight without being strained or over arched. You can close your eyes. And we begin by offering loving kindness to ourselves. By silently repeating phrases like, “may I be safe, be happy, be healthy, live with ease.” Repeat them with enough space and enough silence so that it is a rhythm that pleasing to you. This is like the song of the heart. It’s just one phrase at a time with all of your intention gathered behind that one phrase. “May I be safe, be happy, be healthy, live with ease.”

1.40 – If you find your attention wandering, don’t worry about it. You can simply let go of distractions and begin again. Feelings, thoughts, or memories may come and go. You can allow them to arise and pass away. Here the anchor is the repetition of these phrases. May I be safe, be happy, be healthy, live with ease.

3.09 – And you can call to mind someone who has helped you. They’ve been good to you or kind to you. Or maybe you’ve never met them but they’ve inspired. If someone like that comes to mind, bring them here. You can get an image of them. Say their name to yourself. Get a feeling of their presence and offer the phrases of loving kindness to them. You can wish for them just what it is you’ve wished for yourself. Maybe it’s an adult, maybe it’s a child, maybe it’s an animal. Someone, when you think of them, you smile. If there is someone like that that comes to mind, you can begin directing the phrases to them. May you be safe, be happy, be healthy, live with ease.

4.33 – Even if the words don’t fit totally it doesn’t matter. They’re the conduits of your heart. They’re the vehicles for connection. “May you be safe, be happy, be healthy, live with ease.”

5.53 – Call to mind someone who’s hurting, who’s having a difficult time right now. You can get an image of them. Say their name to yourself. Get a feeling of their presence and offer the phrases of loving kindness to them. May you be safe, be happy, be healthy, live with ease.

7.24 – And if you find your attention wandering, you don’t have to be discouraged. Just gently let go and return your attention to the phrases. One phrase at a time.

7.57 – You can call to mind someone you might encounter just now and then. Maybe a neighbor, maybe someone you see when you walk your dog. Perhaps you don’t even know their name. But you can picture them, get a feeling of their presence. You might not know much or anything about them. You don’t know their story. We can know that this person wants to be happy just as we do. That they are vulnerable to pain and loss just as we are. And we can wish them well. May you be safe, be happy, be healthy, live with ease.

9.39 – And then you can offer your well wishes, the force of loving kindness to all beings everywhere: all people, all creatures, all those in existence, known and unknown, near and far. May all beings be safe, be happy, be healthy, live with ease.

10.59 – And when you feel ready you can open your eyes.

11.15 – *Bell*

11.26 And as you go throughout your day you have various encounters, conversations. If you’re speaking to someone else see if you can actually gather your attention and be there, to listen. If you meet a stranger, go to the super market, you interact with the check-out person, see if you can pay attention to the quite fully. Wishing for their happiness, their well-being, just as you would wish for yourself.

**Breath Awareness (12 minutes 13 seconds)**

0.0 – 0.11: *Bell*

0.11 – To begin with, you can sit comfortably and relax. You don’t have to feel self-conscious as though you are about to do something special or weird. Just be at ease. It helps if your back can be straight without being strained or over arched. You can close your eyes. You can deliberately take three or four deep breathes. Feel the air as it enters your nostrils, fills your chest and abdomen, and then flows out again. Just feel the breath as it happens without trying to change it or improve it.

1:08 – Notice where you feel your breath most vividly, most predominantly. Maybe it’s the in and out movement of air at the nostrils. Maybe it’s the rising falling movement of the chest or the abdomen. You can find that place, bring your attention there and just rest. Rest your attention lightly; the way a butterfly rests on a flower. And see if you can feel just one breath. Without concern for what’s already gone by. Without leaning forward for even the very next breath. Just this one.

2.02 - Be Aware of the sensations of the breath. If you’re at the nostrils, for example, you may feel tingling, vibration, warmth, coolness. If you’re at the abdomen or the chest, you may feel movement, pressure, stretching, release. You don’t need to name these sensations. But feel them

2.45 – Just let your attention rest on the feeling of the natural breath. One breath at a time.

3.31 – To help support the awareness of the breath you might want to experiment with silently saying to yourself, “in, out,” or perhaps, “rising, falling.” But very quietly. So that what you are resting your attention on is really the sensation, the feeling of the breath. Using the word just as a support.

4.12 – And if thoughts or images or emotions or sensations should arise, and they’re not strong enough to actually take you attention away from the breath. Just let them flow on by. You’re breathing

4.26 – They can come and go. Without you chasing after them to hold on or push away.

5.13 – But if something arises that is strong enough to take your attention away. Or you fall asleep. Or you get lost in thought. Don’t worry about it. The moment that we realize that our attention has wandered is the magic moment of the practice. Because that’s the moment we have the chance to be really different. So that instead of judging ourselves and berating ourselves and condemning ourselves, we can be gentle with ourselves, we be kind. Simply let go. See if you can begin again. Bring your attention back to the feeling of the breath.

6.41 – And if you have to let go and begin again, over and over again. Its fine. That’s the practice. You don’t have to get mad at yourself for having a thought. You don’t have to evaluate its content. Just realize you’ve been lost, gently let go, and bring your attention back.

7.36 – If you find yourself getting distracted by thoughts, you can think of those thoughts as clouds moving across a vast sky. The clouds aren’t’ the sky. And actually the sky remains unchanged by them. However many there are. Whatever they look like. Rather than get attached to any one thought. You can just let your thoughts float by. Just like clouds moving through the sky.

8.36 – If you feel sleepy, you can sit up straighter. Maybe open your eyes if they’ve been closed. Again, take a few deep breaths and then return to breathing naturally.

9.04 – You don’t need to control the breath or make it different from the way it is. Simply be with it. Feel the beginning of the in breath, and the end of it. Beginning of the out breath, and the end of it.

10.08 – If you realize you’ve been distracted or you’ve fallen asleep, don’t tell yourself you are weak or undisciplined. Don’t give up in frustration but practice letting go and beginning again.

11.22 – And when you feel ready, you can open your eyes or lift your gaze.

11.31 – *Bell*

11.40 – As you finish this meditation session, reflect on the fact that you can bring some of these same qualities of concentration that you just experienced: presence, calm observation, willing ness to start over, gentleness, forgiveness, to the next activity you do at home, at work, among friends, among strangers.

**Gratitude (12 minutes 5 seconds)**

0 - In our lives, there are many things both large and small for which we may feel grateful. Think back over the past day, past week, past year, and throughout your life and write down all of the things in your life you feel grateful for, both large and small. You will have 5 minutes to make your list so take yourtime to think carefully and deeply about those things for which you feel grateful and put down anything that comes to mind. When your 5 minutes are complete, you will receive another prompt. Please continue to add to your list until you hear the next set of instruction.

5.00 – Okay, good. You can stop adding to your list now. Continue listening to the audio file and you will hear instructions that will promt you to reflect more on the items in your gratitude list.

5.42 – *Bell*

5.53 – To begin with, you can sit comfortably and relax. You can close your eyes. Or you can look at your gratitude list if it’s helpful. Bring to mind a person, event or experience associated with the items on your list. Try to recall and allow the experience of sincere heartfelt gratitude associated with the event to be experienced now. Really open to allowing yourself to feel grateful. To feel the gratitude associated with what’s on your list.

7.24 – And you can call to mind someone who has helped you. Recall the items on your list. Really picturing them and the events surrounding them. See if you can stay with this feeling of gratitude. Really feeling grateful.

8.17 – Reflect on all there is in life that you feel grateful for. Connecting with the sincere, heartfelt feelings of thankfulness. You can call to mind a particular situation or event for which you are particularly thankful. Reflect on it and the people or details surrounding it. Form an image in your memory. See if you can really stay with the feeling of gratitude associated with these people, events, or things.

9.39 – Think back to your gratitude list and all of the things for which you feel grateful. If it helps, focus on one powerful item and why it makes you feel the way you do. Or go through the list, recounting all that there is.

10.32 – Reflect on an experience that inspired gratitude, or awe, or appreciation. Reflect on it and the people or details surrounding it. Form and image in your memory.

11.21 – And when you feel ready, you can open your eyes.

11.47 – As you finish this practice, keep in mind that you can bring some of these same feelings of gratitude to the next activity you do. Whether at home, at work, among friends or people you don’t know.

**Attention Control Script (12 minutes 7 seconds)**

0.0 – 0.11: Bell

0.11 - The places where we live can be very important to us. Think about the place where you live. Your home. If you’ve recently moved and haven’t lived in the same place for at least 6 months, think about the place where you most recently lived for at least 6 months and called home. Try to visualize this place in your mind forming a detailed picture, and write down a detailed description. Start from the entrance and move your way slowly through the house or building until you get to the room that you sleep in. If you want, you can go through the entire house. You will have 5 minutes to write your description, so take your time. When your five minutes are complete, you will receive another prompt. Please continue to add to your description for the entire five minutes until you hear the next set of instructions.

5:00 - OK, good, you can stop adding to your description now. Continue to listen to the audio file and you’ll hear instructions that will prompt you to reflect more on your description.

5:42 – Bell

5.53 – To begin with, you can sit comfortably and relax. You can close your eyes. Or you can look at the description of your home if it’s helpful. Bring to mind a room in your home that you described in great detail. Try to visualize the room and imagine yourself in the room, picturing it in as great of detail as possible, for example, the color of the walls, the furniture in the room, the windows, lighting and anything else that captures your attention. Allow yourself to experience being in that room as if you were there now.

7:24 - And you can call to mind a different room in your home. Recall the details in your description. Really picturing them and the room surroundings. Imagining yourself in those surroundings.

8.17 – Imagine yourself in your favorite room, your favorite place in your home. Put yourself in that place and try to imagine you are really there. Form an image in your memory. See if you can really stay with the feeling of being in your favorite place at home.

9.39 – Think back to your description of your home and imagine that you are there. If it helps, you can imagine yourself in another space inside of your home. Or imagine yourself walking through your home. Relive the experience of being in and moving around your home.

10.32 – Reflect on and visualize a different place in your home. Imagine being there and form an image in your memory of your surroundings, the details, the feeling of the place.

11.21 – And when you feel ready, you can open your eyes.

11.47 As you finish this practice, keep in mind that you can bring some of these same feelings and the experience of being in your home to the next activity you do. Whether at work, among friends or people you don’t know.
